# A Novel Partitivirus in the Hypovirulent Isolate QT5-19 of the Plant Pathogenic Fungus *Botrytis cinerea*

**DOI:** 10.3390/v11010024

**Published:** 2019-01-03

**Authors:** Md Kamaruzzaman, Guoyuan He, Mingde Wu, Jing Zhang, Long Yang, Weidong Chen, Guoqing Li

**Affiliations:** 1 The Key Laboratory of Plant Pathology of Hubei Province and The State Key Laboratory of Agricultural Microbiology, Huazhong Agricultural University, Wuhan 430070, China; kamaru.m@webmail.hzau.edu.cn (M.K.); hgygh2015@163.com (G.H.); mingde@mail.hzau.edu.cn (M.W.); zhangjing1007@mail.hzau.edu.cn (J.Z.); yanglong@mail.hzau.edu.cn (L.Y.); 2 U. S. Department of Agriculture, Agricultural Research Service, Washington State University, Pullman, WA 99164, USA; w-chen@wsu.edu

**Keywords:** *Botrytis cinerea*, hypovirulence, partitivirus, conidiogenesis, sclerogenesis

## Abstract

A pink isolate (QT5-19) of *Botrytis cinerea* was compared with three gray isolates of *B. cinerea* for growth and morphogenesis on potato dextrose agar (PDA), and for pathogenicity on tobacco. A double-stranded (ds) RNA mycovirus infecting QT5-19 was identified based on its genome feature and morphology of the virus particles. The results showed that QT5-19 grew rapidly and established flourishing colonies as the gray isolates did. However, it is different from the gray isolates, as it failed to produce conidia and sclerotia asthe gray isolates did. QT5-19 hardly infected tobacco, whereas the gray isolates aggressively infected tobacco. Two dsRNAs were detected in QT5-19, dsRNA 1 and dsRNA 2, were deduced to encode two polypepetides with homology to viral RNA-dependent RNA polymerase (RdRp) and coat protein (CP), respectively. Phylogenetic analysis of the amino acid sequences of RdRp and CP indicated that the two dsRNAs represent the genome of a novel partitivirus in the genus *Alphapartitivirus*, designated here as Botrytis cinerea partitivirus 2 (BcPV2). BcPV2 in QT5-19 was successfully transmitted to the three gray isolates through hyphal contact. The resulting BcPV2-infected derivatives showed rapid growth on PDA with defects in conidiogenesis and sclerogenesis, and hypovirulence on tobacco. This study suggests that BcPV2 is closely associated with hypovirulence of *B. cinerea*.

## 1. Introduction

Mycoviruses (or fungal viruses) are viruses infecting filamentous fungi, yeasts, and oomycetes [1,2,3]. Previous studies demonstrated that mycoviruses widely exist in all major taxonomic groups of fungi and oomycetes [4]. Most mycoviruses have the genomes of RNA, either double-stranded RNA (dsRNA) or single-stranded RNA (ssRNA), whereas a few mycoviruses have the genomes of single-stranded DNA or ssDNA [5]. The mycoviruses in the families *Chrysoviridae*, *Megabirnaviridae*, *Patitiviridae*, *Reoviridae*, and *Totiviridae* have the dsRNA genomes, which are encapsidated within the coat proteins (CP), thereby forming virus particles. On the other hand, the mycoviruses in the other families such as *Hypoviridae* and *Narnaviridae* have the positive ssRNA (+ssRNA) genomes, which are unencapsidated without formation of virus particles [6,7]. Recently, a few mycoviruses with the genomes of negative ssRNA (−ssRNA) have been identified in a few plant pathogenic fungi, including *Botrytis cinerea* [8,9], *Erysiphe pisi* [10], *Fusarium graminearum* [11], and *Sclerotinia sclerotiorum* [12].

Partitiviruses have been identified in fungi, as well as in plants and protozoa [1,13]. The genome of each partitivirus possesses two dsRNA segments (dsRNA 1 and dsRNA 2), which have the length ranging from 1300 bp to 2500 bp. Each dsRNA segment contains one open reading frame (ORF) on the positive RNA strand. The ORF in dsRNA 1 usually codes for RNA-dependent RNA polymerase (RdRp), whereas the ORF in dsRNA 2 usually codes for coat protein (CP), thereby forming virus particles with the isometric shape of 25 to 40 nm in diameter [13,14].

*Botrytis cinerea* Pers.: Fr. (teleomorph *Botryotinia fuckeliana* (de Bary) Whetzel) is a ubiquitous plant pathogenic fungus [15,16]. It causes gray mold disease on more than 1400 plant species, including many economically important crops such as cucumber (*Cucumis sativus*), strawberry (*Fragaria* × *ananassa*), table grapes (*Vitis vinifera*), and tomato (*Lycopersicon esculentum*) [17]. Substantial economic losses can be caused by *B. cinerea* in these crops before or after harvest under cool-to-temperate and humid conditions [18]. *B. cinerea* is a typical necrotrophic fungus and it usually uses an arsenal of chemical weapons (i.e., cell-wall-degrading enzymes and phytotoxic metabolites) to infect plant tissues [19]. Thus far, cultivars/varieties highly resistant to infection by *B. cinerea* are not available in the crops mentioned above. Control of *B. cinerea* in these crops largely depends on the repeated use of fungicides in China as well as in many other countries [16]. The chemical control is effective when appropriate fungicides are applied to plant tissues in a timely manner. However, some unintended side effects, including fungicide residues in crop produces, pollution to environments, development of fungicide-resistant individuals of *B. cinerea*, may arise due to repeated use of fungicides, thereby causing public concerns over use of the fungicides for control of *B. cinerea*. Therefore, it is necessary to explore alternative measures to control *B. cinerea*, including biological control using ‘natural enemies’ such as mycoviruses.

Studies on mycoviruses infecting *B. cinerea* dated back to the middle of the 1990s, when Howitt and colleagues (1995) first observed virus-like particles (VLPs) in *B. cinerea* and detected dsRNA molecules in this fungus [20]. Since then, many researchers showed interests in identification of mycoviruses in different isolates of *B. cinerea* [8,21,22,23,24,25,26,27,28,29,30]. Several mycoviruses from *B. cinerea* have been characterized at the genome level, including Botrytis virus F (BVF) [21], Botrytis virus X (BVX) [22], Botrytis cinerea endornavirus 1 (BcEV1) [27], Botrytis cinerea hypovirus 1 (BcHV1) [28], Botrytis cinerea fusarivirus 1 (BcFV1) [28], Botrytis cinerea mitovirus 1 (BcMV1) [24], Botrytis cinerea mymonavirus 1 (BcMyV1) [9], Botrytis cinerea negative-stranded RNA virus 1 (BcNSRV-1) [8], Botrytis ourmia-like virus (BOLV1) [26], Botrytis cinerea partitivirus 1 (BcPV1) [31], Botrytis cinerea RNA virus 1 (BcRV1) [32], Botryotinia fuckeliana partitivirus 1 (BfPV1) [33], and Botryotinia fuckeliana totivirus 1 (BfTV1) [34]. Among these mycoviruses, BcEV1, BcFV1, BcHV1, BcMV1, BcMyV1, BcNSRV-1, BcPV1, BcRV1, and BOLV1 were found to be closely associated with hypovirulence of *B. cinerea* [8,9,23,26,28,31,32]. However, none of these mycoviruses has been found to have potential as a biocontrol agent for control of the diseases caused by *B. cinerea*. Two factors might be responsible for this situation. First, the mycoviruses have limited horizontal transmissibility possibly due to hyphal (vegetative) incompatibility between the mycoviruses-containing and mycoviruses-free individuals. Second, the mycovirus-infected *B. cinerea* isolates usually grow poorly [23,35] and they may have lower competitive ability than the virulent isolates. Therefore, potential mycoviruses for successful control of *B. cinerea* need to overcome these limitations.

A pink-colored isolate (QT5-19) of *B. cinerea* was obtained from a diseased fruit of tomato collected in Shaanxi Province of China. Our preliminary study showed that QT5-19 was almost non-pathogenic on many plants, including tomato, tobacco and oilseed rape (*Brassica napus*), suggesting that this isolate was debilitated in pathogenicity or virulence. It is suspected that some mycoviruses may exist in this particular isolate. Therefore, this study was conducted to characterize the mycovirus in QT5-19.

## 2. Materials and Methods

### 2.1. Fungal Isolates and Cultural Conditions

Five fungal isolates of *B. cinerea* (08168, B05.10, QT5-19, RoseBC-3, XN-1) were used in this study. QT5-19 was isolated from a diseased fruit of tomato collected from Shaanxi Province of China. The identity of QT5-19 as *B. cinerea* was confirmed both by PCR detection using the *B. cinerea*-specific PCR primers reported by [36], and by analysis of the sequence of ITS-rDNA [37] region. Four other *B. cinerea* isolates (08168, B05.10, RoseBC-3, XN-1) were obtained from our previous collection and their origin was described in related reports [37,38,39,40,41]. All the fungal isolates were incubated at 20 °C on potato dextrose agar (PDA) in the dark for 2 to 3 days to determine radial growth rates and for 20 days to determine sclerotial production [42].

Production of the pink color in the colonies of QT5-19 suggests that this isolate may produce the pink pigment bikaverin [43]. In order to confirm this hypothesis, expression of six bikaverin biosynthesis-related genes (*bcbik1* to *bcbik6*) in QT5-19 (pink color) and B05.10 (gray color) was detected by RT-PCR (Figure S1) using the total RNA of each isolate as template and the specific PCR primer sets listed in Table S1. To confirm correctness of the *bcbik1* gene in QT5-19, the RT-PCR product of *bcbik1* from QT5-19 was purified from the agarose gel, cloned into *E. coli* DH5α and sequenced, and the resulting cDNA sequence was compared with the DNA sequence of *bcbik1* in *B. cinerea* isolate 1750 (GenBank Acc. No. HE802550) (Figure S2).

### 2.2. Determination of Pathogenicity

Seeds of tobacco (*Nicotiana benthamiana*) were sown in plastic pots containing Organic Culture Mix containing 2% to 5% of N + P_2_O_5_ + K_2_O (*w:w*, N:P_2_O_5_:K_2_O = 1:1:1) (Zheng Jiang Pei Lei Organic Manure Manufacturing Co., Ltd., Zhengjiang City, Jiangsu Province, China). The pots were placed in a growth room at 20 °C for 40 days under the lighting regime of 12-h light/12-h dark and watered as required. Fully-expanded leaves were detached from the plants and placed on moist paper towels in a plastic tray (45 × 30 × 2.5 cm, length × width × height) with their adaxial surface facing up. Mycelial agar plugs (5 mm in diameter) from the margin area of a two-day-old PDA culture of an investigated isolate of *B. cinerea* were placed on the leaves, one mycelial agar plug per leaf and six leaves (as six replicates) for each isolate. The tray was covered with a 0.1-mm-thick transparent plastic film (Gold Mine Plastic Industry Ltd., Jiang Men City, China) to maintain high humidity and placed in a growth chamber at 20 °C for three days (12-h light/12-h dark). Diameter of the leaf lesion formed around each mycelial agar plug was measured.

Additionally, isolates QT5-19 and B05.10 were tested for infection of apple (*Malus demestica*), table grapes (*Vitis vinifera*), tomato (*Lycopersicon esculentum*), cucumber (*Cucumis sativus*), oilseed rape (*Brassica napus*), and strawberry (*Fragaria* × *ananassa*) (cultivars unknown for all of the investigated crops). Fruits of apple, table grapes and tomato purchased from a local supermarket, and leaves of cucumber, oilseed rape, and strawberry detached from adult plants were placed on moist paper towels in plastic trays with three replicates for each of the two isolates, three fruits or three leaves per replicate, one agar plug on each fruit or leaf. The trays were individually covered with transparent plastic films and placed in the growth chamber (20 °C) for three days. Lesion diameter on each fruit or leaf around each mycelial agar plug was measured (Figure S3).

### 2.3. Extraction of dsRNAs

Isolate QT5-19 was inoculated on cellophane films (CF) placed on PDA in Petri dishes as the CF-PDA cultures. The cultures were incubated at 20 °C in the dark for three days. The mycelial mass was harvested from the cultures and ground to a fine powder in liquid nitrogen. DsRNAs were extracted from the mycelial powder using the cellulose (CF-11) chromatography method described by Wu and colleagues (2007, 2010, 2012). The extract was digested with RQ1 RNase-free DNase (Promega, Madison, WI, USA) for elimination of DNA contamination and with S1 nuclease (TaKaRa) for elimination of single-stranded RNAs (ssRNAs) in the extracts. The dsRNAs were detected by agarose gel (0.7% or 1.0%, *w*/*v*) and visualized by staining with Biosharp^®^ SuperRed/GelRed (Guangzhou Sai Guo Biotechnol. Co., Ltd., Guangzhou, China). 

### 2.4. cDNA Cloning of the dsRNAs

The dsRNAs extracted from isolate QT5-19 were purified from an agarose gel after electrophoresis using Axygen^®^ DNA gel extraction kit (Axygen^®^ Scientific Inc., Union City, CA, USA). The pure dsRNAs were ligated with the adaptor 110a (Figure S4, Table S1) at the 3′-terminus using T4 RNA ligase (Promega, Madison, WI, USA) at 16 °C for 12 h. The adaptor-ligated dsRNAs were purified using AxyPrep^TM^ PCR cleanup kit (Axygen Scientific Inc., Union City, CA, USA). For synthesis of cDNAs, the adaptor-ligated dsRNAs (10 µL) were mixed with 1 µL oligonucleotide primer RC110a (complementary to the adaptor 110a, Supporting Information Table S1), 1 µL dNTPs (10 mmol/L), 1 µL dimethyl sulfoxide (DMSO) and 2 µL DEPC-H_2_O. The mixture was denatured at 98 °C and chilled on ice for 120 s. Then, PrimeScript II reverse transcriptase (TaKaRa Biotechnol. Co., Ltd., Dalian, China) and 5× PrimeScript II buffer (TaKaRa) were added to the mixture and the reverse transcription was done at 42 °C for 1.5 h. The full-length cDNAs were PCR amplified in S1000^TM^ Thermal Cycler (BIO-RAD Laboratories Inc., Hercules, CA USA) using the synthetic cDNAs as templates in the presence of the primer RC110a [44]. The cDNA cloning was repeated five times to guarantee sequence accuracy. All the PCR products were separated by 1% (*w*/*v*) agarose gel electrophoresis, followed by gel purification of the cDNA molecules and ligation of the cDNAs into the pMD18-T vector (TaKaRa), which was then transformed into *E. coli* DH5α (TaKaRa) for proliferation and sequencing.

### 2.5. Analysis of the Mycoviral Genome

The open reading frame (ORF) in the full-length cDNA sequence of each mycoviral dsRNA was identified using the ORF Finder program in GenBank at the website of NCBI (https://www.ncbi.nlm.nih.gov/) with the standard codon usage. The conserved domains and motifs in each ORF were identified by comparison with those in related genomes deposited in the public databases of GenBank and PROSITE (http://prosite.expasy.org/). Multiple sequence alignment of the amino acid sequence for RNA-dependent RNA polymerase (RdRp) was performed using the T-Coffee server in the website (http://tcoffee.crg.cat/apps/tcoffee). The gaps were manually removed using the CLUSTAL_W program. Nucleotide (nt) and amino acid (aa) sequences of the previously-reported partitiviruses and related members were retrieved from GenBank (Table S2). They were used for comparative analysis and for inference of the phylogenetic relationship of the mycovirus in QT5-19. Phylogenetic trees were generated based on the amino acid sequences of RNA-dependent RNA polymerase (RdRp) and coat protein (CP) using the Maximum-Likelihood (ML) method in the MEGA software version 7.0 [45]. They were individually tested with the bootstrap value of 1000 to ascertain the reliability of a given pattern of the ML trees. Potential secondary structures at the 5′- and 3′-termini of each dsRNA were predicted using RNA Structure version 5.8.1 [46].

### 2.6. Purification of Virus Particles

Isolate QT5-19 was incubated on CF-PDA at 20 °C for eight days. The mycelial mass (~8 g) harvested from the CF-PDA cultures was ground to fine powder in liquid nitrogen and the powder was suspended in 200 mL phosphate-buffered saline (PBS) (0.1 mol/L, pH 7) amended with 6 mL Triton-X 100. The slurry was further homogenized in a glass homogenizer for 30 min. The homogenate was transferred to a 200-mL-plastic tube and centrifuged at 10,000× *g* for 20 min to remove the hyphal debris. The supernatant was transferred to another plastic tube and centrifuged at 119,000× *g* under 4 °C for 2 h. The supernatant was discarded and the pellet was suspended in 0.1 mol/L PBS and the suspension was centrifuged at 16,000× *g* for 20 min to remove large particles. The supernatant was loaded in a centrifuge tube containing sucrose with the concentration gradient ranging from 10% to 50% (*w*/*v*) and centrifuged at 70,000× *g* under 4 °C for 2 h. The fractions were carefully collected by punching the centrifuge tube with a clean sterilized syringe and separately measured for the existence of the virus partials by detection of the abundance of the dsRNAs by agarose electrophoresis. The fraction containing the virus particles was suspended in 50 μL PBS (50 mmol/L, pH 7.0). The virus particles were stained with phosphotungstic acid (20 g/L, *w*/*v*, pH 7.4) and observed under transmission electron microscope (TEM). The dsRNAs from the viral particles were extracted with phenol chloroform isoamyl alcohol (25:24:1, *v:v:v*), and detected by agarose gel electrophoresis. Meanwhile, the purified virus particles in PBS were boiled and denatured at 100 °C for 5 min, and the solution was centrifuged at 12,000 r/min for 2 min. The resulting supernatant was mixed with 5× Tris-glycerol loading buffer (250 mM Tris·HCl, 10% SDS, 30% glycerol, 10 mM DTT, 0.05% bromophenol blue, pH 6.8) at the volume ratio of 1:5. The mixture was the loaded in a 10% SDS-PAGE gel. The pre-stained protein ladder, PageRuler^TM^ (Thermo Fisher Scientific Inc., Watham, MA USA) was loaded in the gel as the molecular weight standard. After electrophoresis (120 V, 2.5 h), the gel was stained with Coomassie brilliant blue R-250.

### 2.7. RT-PCR Detection of the Mycovirus in QT5-19 and Northern Blotting

Total RNA was extracted from the mycelia of the investigated isolates of *B. cinerea* using TaKaRa RNAiso Plus Total RNA Extraction Reagents (TaKaRa). It was used in reverse transcription-PCR (RT-PCR) for detection of the mycovirus in QT5-19 and other isolates with the specific primer pairs BcPVRd-F/BcPVRd-R and BcPVCP-F/BcPVCP-R targeting the ORFs for RdRp and CP, respectively (Table S1). Meanwhile, a specific primer pair Act-F/Act-R targeting the actin gene of *B. cinerea* was used as a reference gene in RT-PCR [32].

Northern blotting was carried out to confirm authenticity of the full-length cDNAs from the dsRNAs in isolate QT5-19. It was done using the procedure described in our previous studies [23,35]. Two DNA probes targeting the two ORFs of the dsRNAs coding for RdRp and CP were generated by PCR with the two cDNAs as templates and the specific primer pairs RdRp-F/RdRp-R and CP-F/CP-R (Figure S5, Table S1).

### 2.8. Horizontal Transmission of the Mycovirus in QT5-19

Horizontal transmission of the mycovirus from QT5-19 (donor) to *B. cinerea* isolates 08168, B05.10, RoseBc-3, and XN-1 (recipients) was done on PDA in Petri dishes (9 cm in diameter) using the pair culturing technique described in our previous study [44]. Three derivative isolates were obtained from the colonies of each recipient in the four-day-old pair-cultures. These derivatives were easily distinguished from QT5-19, as they formed gray-colored colonies rather than pink-colored colonies like those of QT5-19. Five representative derivatives (08168T, B05.10T, RoseBc-3T, and XN-1T from 08168, B05.10, RoseBc-3, and XN-1 in the pair-cultures, respectively) were tested for the presence of the mycovirus by dsRNA profiling and RT-PCR with the mycovirus-specific primer pairs RdRp-f1/RdRp-r1 and CP-f1/CP-r1 (Table S1). Meanwhile, these derivatives, as well as their parents, were tested for growth rates and production of conidia/sclerotia on PDA, and for pathogenicity on tobacco leaves using the methods described above.

### 2.9. Elimination of the Mycovirus in QT5-19

Four trials (protoplast regeneration, hyphal tipping, thermal treatment, and ribavirin treatment) were done to eliminate the mycovirus in isolate QT5-19. In the protoplast regeneration trial, the two-day-old mycelial mass of QT5-19 from the CF-PDA cultures was harvested and blended to hyphal fragments in sterile distilled water (10 g fresh mycelia in 100 mL water). The resulting hyphal fragment suspension (HFS) was inoculated in a 250-mL-Erlenmeyer flask containing PDB at the volume ratio of 1:20 (HFS:PDB), and incubated overnight at 150 rpm and 20 °C. The culture was centrifuged at 2000× *g* and 4 °C for 10 min to collect the mycelial pellet, which was then suspended in 1 mol/L sorbitol amended with 1% (*w*/*v*) Lysing Enzyme L2265 (Sigma-Aldrich Chemical Company Ltd., St. Louis, MO, USA) and 0.1% snailase (*w*/*v*) (Solarbio^®^ Life Sciences, Beijing, China). The suspension was gently shaken at 30 °C for 3 h to release the protoplasts and the resulting mixture was filtered through double-layered filter papers to remove the hyphal fragments. The protoplasts were collected by centrifugation under 4 °C at 2000× *g* for 20 min, washed twice with 1 mol/L d-sorbitol, and suspended in STC buffer (1 mol/L d-sorbitol, 50 mmol/L Tris-HCl at pH 7.5, 50 mmol/L CaCl_2_). The suspension was plated on TB3 medium (2 g casein hydrolysate, 5 g yeast extract, 25 g sucrose, 15 g agar, 1000 mL water) and the culture was incubated at 20 °C for seven days for protoplast regeneration and hyphal growth. Fifty-one emerged fungal colonies were individually transferred to fresh PDA, incubated at 20 °C in the dark for 15 days and the colony morphology (color, production of conidia and sclerotia) was observed.

In the hyphal-tipping trial, QT5-19 was incubated on water agar at 20 °C for three days. The cultures were placed under a dissecting light microscope. Hyphal tips (approximately 80 µm in length) in the colony margin were individually cut using a fine needle and transferred to PDA in Petri dishes, one hyphal tip per dish. The resulting cultures were incubated at 20 °C for 15 days and the colony morphology was observed. 

In the thermal treatment, QT5-19 was incubated on PDA at 30 °C for 30 days. Mycelial agar plugs (~3 mm in diameter) were removed from the margin area of the cultures. They were individually transferred to fresh PDA in Petri dishes, one mycelial agar plug per dish. The resulting cultures were incubated at 30 °C for 30 days and each culture was sub-cultured again on PDA at 30 °C for 30 days. Finally, the cultures were transformed to PDA and incubated at 20 °C for 15 days, and the colony morphology of each subculture was observed.

In the ribavirin treatment, QT5-19 was inoculated in PDA amended with ribavirin at 0.5, 1.0, or 1.5 mg/mL. The cultures were incubated at 20 °C for seven days. Then, mycelial agar plugs were removed from the cultures of each ribavirin treatment and transferred to new PDA containing the same concentrations of ribavirin and the subcultures were incubated at 20 °C also for seven days. This process was repeated two more times. Finally, the subcultures were incubated on PDA alone at 20 °C for 15 days and the colony morphology of each subculture was observed.

Seventy out of the 111 derivative isolates of QT5-19 from the above-mentioned trials were arbitrarily selected (15, 15, 20, and 20 isolates from hyphal tipping, thermotherapy, chemotherapy and protoplast regeneration, respectively). They were incubated on PDA at 20 °C in the dark for observation of the colony morphology and for detection of the mycovirus by dsRNA profiling and RT-PCR with the specific primers RdRp-f1/RdRp-r1 and CP-f1/CP-r2 (Table S1, Figure S6).

### 2.10. Statistical Analysis

Data in each experiment or assay were analyzed using the procedure of Analysis of Variance (ANOVA) in the SAS software (SAS Institute, Cary, NC, USA, v. 8.0, 1999). Treatment means about mycelial growth rates on PDA in the colony morphology-characterization trials, lesion diameters on leaves of tobacco in the pathogenicity tests were separated using the least significant difference (LSD) test at *α* = 0.05. Means about mycelial growth rates and numbers of sclerotia per dish on PDA, and leaf lesion diameters on tobacco and between each BcPV2-transfected derivative (08168T, B05.10T, RoseBc-3T, XN-1T) and its parental isolate (08168, B05.10, RoseBc-3, XN-1) were compared using Student’s *t* test at *α* = 0.05 or 0.01.

## 3. Results

### 3.1. Cultural Characteristics

**Figure 1:**
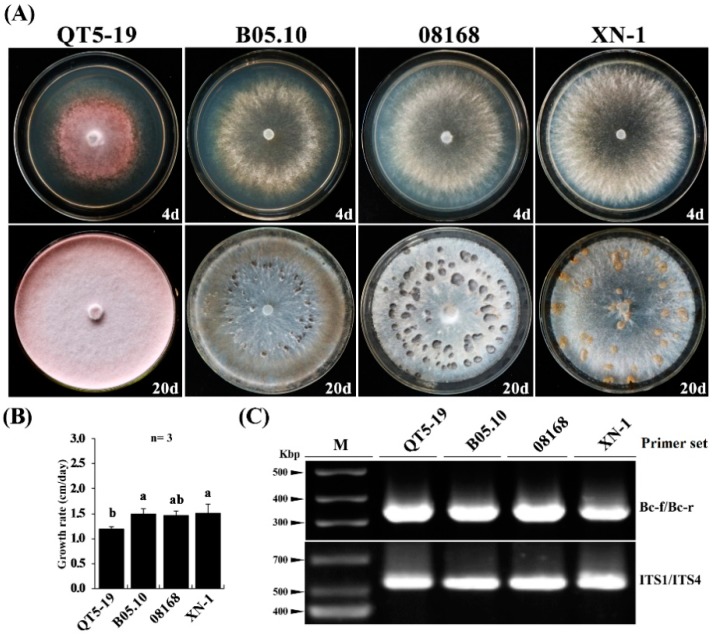
Cultural characteristics of *Botrytis cinerea* isolates on potato dextrose agar. (**A**) Four-day-old (top) and 20-day-old cultures (bottom) of isolates QT5-19, 08168, B05.10, and XN-1 (20 °C). Note difference in colony color, pink for QT5-19, whereas gray for the other three isolates. Note also difference in sclerotial color, orange sclerotia produced by isolate XN-1, whereas black sclerotia produced by isolates 08168 and B05.10; (**B**) A histogram showing average mycelial growth rates of the four isolates. Means ± S.D. (*n* = 4) labeled with the same letters are not significantly different at *p* > 0.05; (C) PCR-based identification of QT5-19 using the *B. cinerea*-specific primer set Bc-f/Bc-r and the universal primer set ITS1/ITS4. Isolates 08168, B05.10, and XN-1 of *B. cinerea* were used as reference.

Isolate QT5-19 grew at an average rate of 1.2 cm/d on PDA at 20 °C and colonized the entire Petri dishes (9 cm in diameter) after incubation for four days. This growth rate was slightly lower than those (1.5 cm/d) in three other isolates of *B. cinerea* (08168, B05.10, XN-1) under the same cultural conditions (Figure 1A,B). QT5-19 formed pink-colored colonies without production of any conidia and sclerotia even after incubation for 20 days. These morphological features differed from those of isolates 08168, B05.10 and XN-1, which formed gray-colored colonies with production of conidia and sclerotia (Figure 1A).

Despite the lack of typical cultural and morphological characteristics, QT5-19 was identified as *B. cinerea* based on two molecular features. First, QT5-19 showed the *B. cinerea*-specific marker, a 327-bp DNA fragment (Figure 1C), in PCR with the *B. cinerea*-specific primers Bc-f/Bc-r reported by Fan and colleagues [36]. Second, the nucleotide sequence of the internal transcribed spacer (ITS)-rDNA region of QT5-19 (GenBank Acc. No. KX822693) is 100% identical to the ITS sequence of *B. cinerea* B05.10 (GenBank Acc. No. CP009808) and 99% identical to the ITS sequence of *B. cinerea* XN-1 (GenBank Acc. No. KT266229).

The pink pigment produced by QT5-19 is possibly bikaverin, as all the six bikaverin biosynthesis-related genes (*bcbik1* to *bcbik6*) were expressed in this isolate (Figure S1). The RT-PCR product of *bcbik1* coding for polyketide synthase (PKS) was cloned from QT5-19 and sequenced. The 679-bp cDNA fragment (GenBank Acc. No. MH747471) was 100% identical to the DNA sequence of *bcbik1* in the bikaverin-producing isolate INRA 1750 of *B. cinerea* (GenBank Acc. No. HE802550) (Figure S2). On the other hand, isolate B05.10 of *B. cinerea* failed to produce bikaverin on PDA. It had expression of five of these six bikaverin biosynthesis-related genes (*bcbik2* to *bcbik6*), but had no expression of *bcbik1* (Figure S1), as it lacks this gene in its genome [43].

### 3.2. Hypovirulence

**Figure 2:**
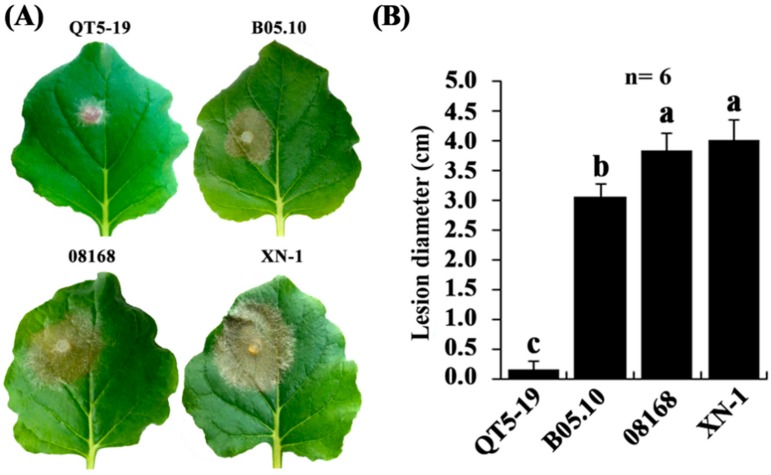
Pathogenicity of *Botrytis cinerea* isolates on tobacco (*Nicotiana benthamiana*). (**A**) Four tobacco leaves inoculated with the mycelia of the isolates QT5-19, 08168, B05.10, and XN-1, respectively (20 °C, 3 dpi). Note no visible lesion formation on the leaf inoculated with isolate QT5-19, whereas formation of large necrotic lesions on the leaves inoculated with the other three isolates; (**B**) A histogram showing average leaf lesion diameters caused by the four isolates. Means ± S.D. (*n* = 6) labeled with the same letters are not significantly different at *p* > 0.05.

Results of the pathogenicity test showed that isolate QT5-19 caused no infection or produced tiny lesions on leaves of tobacco, as well as on leaves of cucumber, oilseed rape, and strawberry, and on fruits of apple, table grapes and tomato at 3 days post-inoculation (dpi) under 20 °C with average lesion diameters ranging from 0.0 to 0.3 cm (Figure 2A,B, Figure S3). In contrast, B05.10, 08168 and XN-1 caused large necrotic lesions on the tobacco leaves with average lesion diameters of 3.1, 3.8, and 4.0 cm, respectively (Figure 2). B05.10 caused large necrotic lesions on apple, cucumber, oilseed rape, strawberry, table grapes, tobacco, and tomato with average lesion diameters ranging from 1.8 cm to 3.0 cm (Figure S3).

### 3.3. The dsRNA Elements 

**Figure 3:**
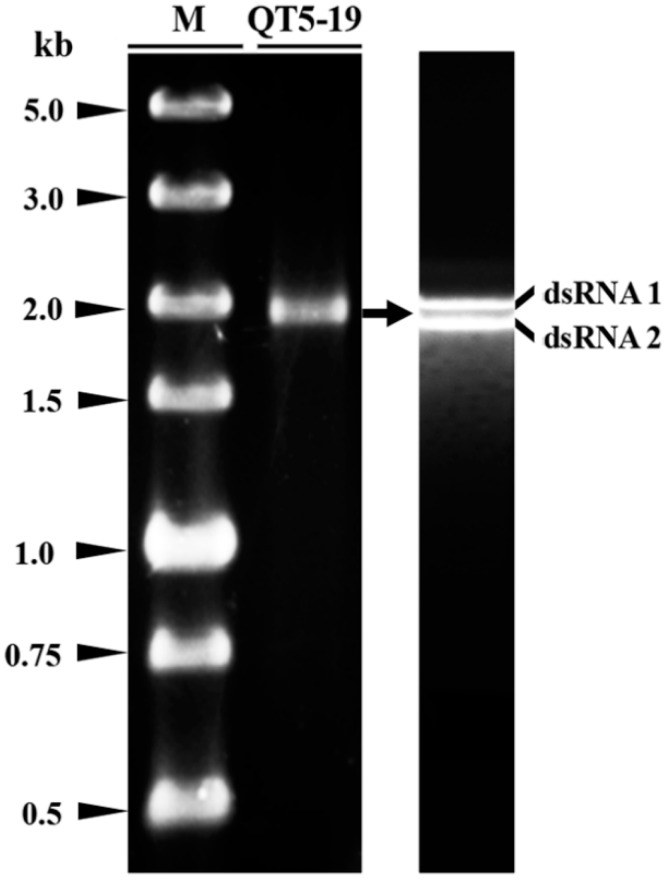
Double-stranded (ds) RNAs in isolate QT5-19 of *Botrytis cinerea*. The electrophoregram on the left was carried out in a 1.0% agarose gel after 1.5-h electrophoresis under room temperature. The electrophoregram on the right was carried out in a 0.7% agarose gel after 15-h electrophoresis at 4 °C. The dsRNAs were treated with RNase-free DNase I and S1 nuclease before electrophoresis. M = DL5000 dsDNA marker (TaKaRa).

Two dsRNA molecules (dsRNA 1 and dsRNA 2) of ~2 kb in size were detected in the extracts of nucleic acids from the mycelia of QT5-19 (Figure 3). In contrast, no dsRNAs were detected in the virulent isolates 08168, B05.10, RoseBc-3, and XN-1 of *B. cinerea*. To determine persistency of the two dsRNAs in QT5-19, repeated attempts using four different approaches (protoplast regeneration, thermotherapy, hyphal tipping, chemotherapy) were made to eliminate the dsRNAs from QT5-19. A total of 111 derivatives were obtained, 15 from hyphal tipping, 15 from thermotherapy, 30 from chemotherapy, and 51 from protoplast regeneration. All these derivatives of QT5-19 formed colonies with the morphology similar to that of the parent QT5-19 (pink color, no production of conidia and sclerotia) on PDA at 20 °C. Seventy derivatives (15 from hyphal tipping, 15 from thermotherapy, 20 from chemotherapy, and 20 from protoplast regeneration) were randomly selected and individually tested for the presence of the dsRNAs by dsRNA profiling and RT-PCR using the total RNA as template and RdRp-f1/RdRp-r1 and CP-f1/CP-r1 as specific primers (Table S1). The results showed that all the selected derivatives contained two dsRNAs with the same size as those in QT5-19 (Figure S6).

### 3.4. Virus Particles

**Figure 4:**
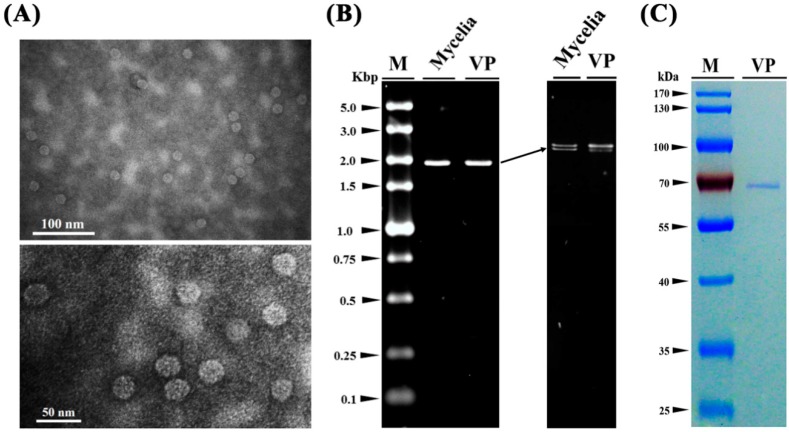
Virus particles isolated from the mycelia of isolate QT5-19 of *Botrytis cinerea*. (**A**) Two transmission electron microscope (TEM) graphs showing the shape and size of the virus particles of the mycovirus in QT5-19; (**B**) Electrophoregrams showing the two dsRNAs extracted from the virus particles (VP) and the mycelia of QT5-19 (Mycelia). The electrophoregram on the right was created in a 1% agarose gel after 1.5-h electrophoresis under room temperature. The electrophoregram on the left was created in a 0.7% agarose gel after 15-h electrophoresis at 4 °C. M = DL5000 marker (TaKaRa); (**C**) An SDS-PAGE (10%) electrophoregram showing the band of the structural protein (coat protein) extracted from the virus particles of the mycovirus in QT5-19. The gel was stained with Coomassie brilliant blue R-250. M = PageRuler™ Prestained Protein Ladder.

Virus particles were isolated from the mycelia of QT5-19 and purified by sucrose density-gradient centrifugation. They are isometric in shape under transmission electronic microscope (TEM) with an average diameter of 36 nm (Figure 4A). They contain two dsRNAs with the same size as those extracted from the mycelia of QT5-19 (Figure 4B) and a protein with the molecular weight of ~68 kDa (Figure 4C).

### 3.5. Identity of the Two dsRNAs

The dsRNAs in QT5-19 were purified from agarose gels after electrophoresis. They were ligated with the adaptor sequence 110a (Figure S4) at the 3′-terminus of each dsRNA. The adaptor-dsRNA molecules were reverse transcribed to cDNAs, which were then used as templates in PCR for cDNA amplification with the primer RC110a complementary to the adaptor 110a (Table S1). The resulting DNA products were separated by agarose gel electrophoresis and the purified fragments were cloned into the pMD18-T vector, which was then transformed into *Escherichia coli* DH5α and sequenced. The full-length cDNAs for dsRNA 1 (GenBank Acc. No. MG011707) and dsRNA 2 (GenBank Acc. No. MG011708) are 1909 and 1883 bp long, respectively. Northern blotting confirmed authenticity of the cDNAs from the dsRNAs in QT5-19 (Figure S5). 

**Figure 5:**
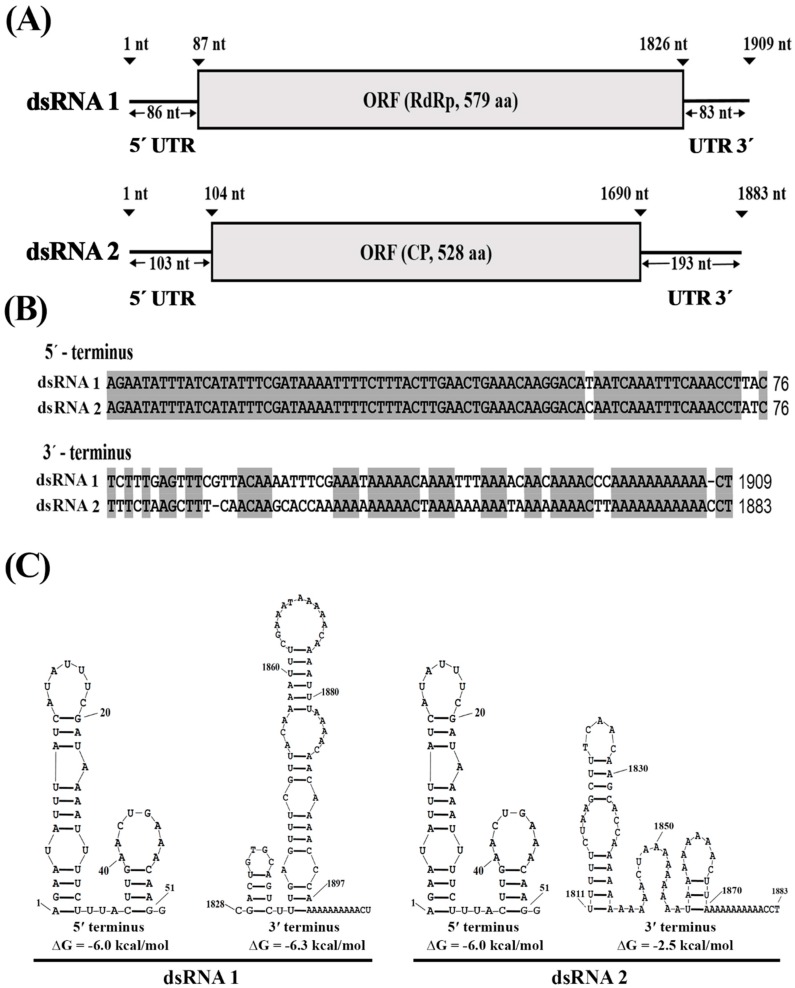
Genome structure of BcPV2. (**A**) A schematic diagram illustrating the segmented dsRNA genome. Each dsRNA has one open reading frame (ORF) flanked by two untranslated regions (UTR) at 3′- and 5′-termini; (**B**) Alignments of the partial nucleotide sequences of the 3′- and 5′-UTRs of dsRNA 1 and dsRNA 2. The identical nucleotides were highlighted in gray color. “-”, missing nucleotides; (**C**) Predicted secondary structures for the terminal regions of dsRNA 1 and dsRNA 2.

Analysis of the nucleotide sequences of the two dsRNAs showed that the coding strand of each dsRNA contains one open reading frame (ORF) flanked by two untranslated regions (UTRs) at 3′- and 5′-termini (Figure 5A). The two dsRNAs shares 63.9% nucleotide identity at the 3′-terminus (72 bp long) and 96.1% nucleotide identity at the 5′-terminus (76 bp long) (Figure 5B). Additionally, the 5′- and 3′-UTRs of each dsRNA were predicted to be able to form stem-loop structures (Figure 5C).

**Figure 6:**
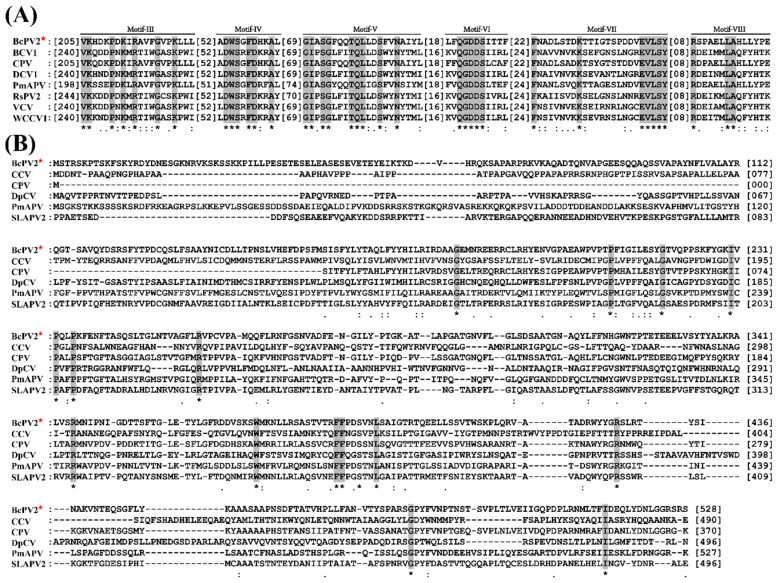
Multiple alignments of the amino acid sequences of RNA-dependent RNA polymerase and coat protein of BcPV2 with other selected members in *Alphapartitivirus*. (**A**) Amino acid sequences of RNA-dependent RNA polymerase (RdRp); and (**B**) amino acid sequences of coat protein (CP). “*”, identical amino acids, “:” and “.” high and low chemically similar amino acids, respectively. Note the five conserved motifs (Motif-III to Motif-VIII) in RdRp and diversified CP sequences among the compared viruses. Abbreviations: BCV1, Beet cryptic virus 1; BcPV2, Botrytis cinerea partitivirus 2; CCV, carrot cryptic virus; CPV, Ceratobasidium partitivirus; DCV1, Dill cryptic virus 1; DpCV, Diuris pendunculata cryptic virus; PmAPV, Powdery mildew-associated partitivirus; RsPV2, Rhizoctonia solani partitivirus 2; SLAPV2, Soybean leaf-associated partitivirus 2; **VCV**, Vicia cryptic virus; WCCV1, white clover cryptic virus 1. The GenBank accession numbers for RdRp and CP of the above-mentioned viruses are listed in Table S2.

The ORF in dsRNA 1 was predicted to encode a polypeptide containing 579 amino acid (aa) residues with the estimated molecular weight of 67.08 kDa. Homology searches in the database of GenBank showed that the 579-aa protein is identical by 13%–76% to the RNA-dependent RNA polymerases (RdRp) encoded by ten partitiviruses, including Botryotinia fuckeliana partitivirus 1 (BfPV1, 13% identity), Ceratobasidium partitivirus (CPV, 76% identity), Flammulina velutipes isometric virus (FvIV, 42% identity), Fusarium solani partitivirus 2 (FsPV2, 41% identity), Grapevine partitivirus (GPV, 53% identity), Powdery mildew-associated partitivirus (PmAPV, 51% identity), Rhizoctonia solani partitivirus 1 (RsPV1, 43% identity), Rosellinia necatrix partitivirus 5 (RnPV5, 40% identity), Sclerotinia sclerotiorum partitivirus S (SsPV-S, 46% identity), and Soybean leaf-associated partitivirus 2 (SLAPV2, 58% identity) (Table 1). It contains six motifs (Motif III to Motif VIII) (Figure 6A), which are also present in the RdRps of some other partitiviruses, such as Beet cryptic virus 1 (BCV1), CPV, Dill cryptic virus 1 (DCV1), PmAPV, Rosellinia necatrix partitivirus 2 (RsPV2), Vicia cryptic virus (VCV), and white clover cryptic virus 1 (WCCV1) (Table 1).

The ORF in dsRNA 2 was predicted to encode a polypeptide containing 528 aa residues with estimated molecular weight of 58.84 kDa. The polypeptide is identical by 10–60% to the coat proteins (CP) encoded by seven partitiviruses, including Arabidopsis halleri partitivirus 1 (AhPV1, 22% identity), BfPV1, Botrytis cinerea partitivirus 1 (BcPV1, 14% identity), CPV (60% identity), PmAPV (31% identity), RnPV2 (23% identity), and SLAPV2 (34% identity), and by 24–31% to the CPs encoded by three plant cryptic viruses, including carrot cryptic virus (CCV, 26% identity), Diuris pendunculata cryptic virus (DpCV, 31% identity) and spinach cryptic virus 1 (SCV, 24% identity) (Table 1). The aa sequence of CP found in this study and its homologs in CCV, CPV, DpCV, PmAPV, and SLAPV2 appeared to be highly divergent (Figure 6B). Therefore, the two dsRNAs in QT5-19 represent the genome of a novel partitivirus in the family *Partitiviridae*, which is hereafter designated as Botrytis cinerea partitivirus 2 (BcPV2).

**Figure 7:**
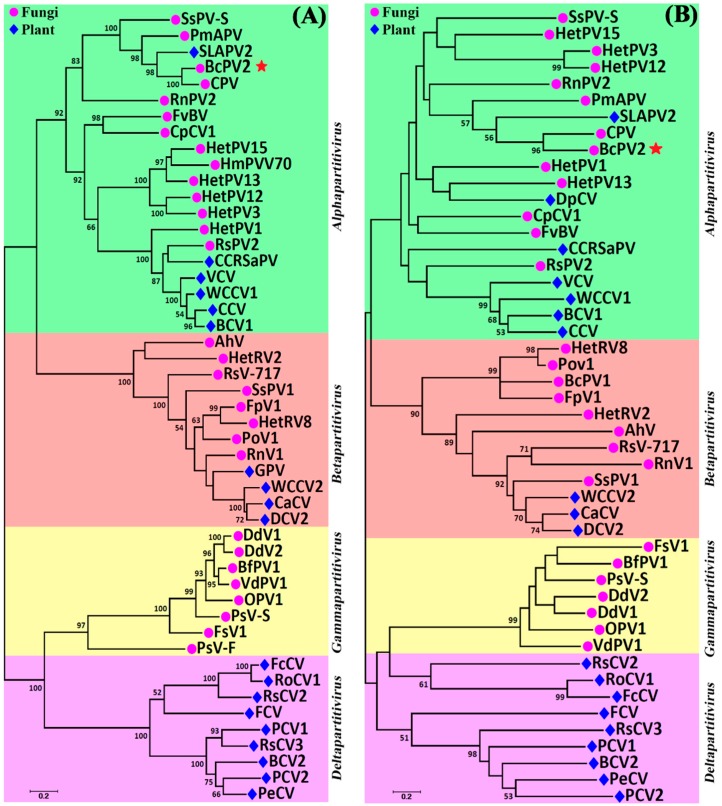
Two phylogenetic trees showing the relationship of BcPV2 with other partitiviruses. The trees were inferred based on amino acid sequences of RdRp (**A**) and CP (**B**) using the Maximum-Likelihood method with bootstrap values determined by 1000 replicates. Bootstrap values higher than 50% are shown in the graphs. The scale bars represent substitutions per nucleotide position. Abbreviations: AhV, Atkinsonella hypoxylon partitivirus; BcPV1, Botrytis cinerea Partitivirus 1; BcPV2, Botrytis cinerea Partitivirus 2; BCV1, Beet cryptic virus 1; BCV2, Beet cryptic virus 2; BfPV1, Botryotinia fuckeliana partitivirus 1; CaCV, Cannabis cryptic virus; CCRSaPV, Cherry chlorotic rusty spot associated partitivirus; CpCV1, Chondrostereum purpureum cryptic virus 1; CPV, Ceratobasidium partitivirus; DCV1, Dill clover cryptic virus 1; DCV2, Dill cryptic virus 2; DdV1, Discula destructiva virus 1; DdV2, Discula destructiva virus 2; FcCV, Fragaria chiloensis cryptic virus; FCV, Fig cryptic virus; FpV1, Fusarium poae virus 1; FsV1, Fusarium solani virus 1; FvBV, Flammulina velutipes browning virus; GPV, Grapevine partitivirus; HetPV1, Heterobasidion partitivirus 1; HetPV12, Heterobasidion partitivirus 12; HetPV13, Heterobasidion partitivirus 13; HetPV15, Heterobasidion partitivirus 15; HetPV3, Heterobasidion partitivirus 3; HetRV2, Heterobasidion partitivirus 2; HetRV8, Heterobasidion partitivirus 8; HmPVV70, Helicobasidium mompa partitivirus V70; OPV1, Ophiostoma partitivirus 1; PCV1, Pepper cryptic virus 1; PCV2, Pepper cryptic virus 2; PeCV, Persimmon cryptic virus; PmAPV, Powdery mildew-associated partitivirus; PoV1, Pleurotus ostreatus virus 1; PsV-F, Penicillium stoloniferum virus F; PsV-S, Penicillium stoloniferum virus S; RnPV2, Rosellinia necatrix partitivirus 2; RnV1, Rosellinia necatrix partitivirus 1; RoCV1, Rose cryptic virus 1; RsCV2, Raphanus sativus cryptic virus 2; RsCV3, Raphanus sativus cryptic virus 3; RsPV2, Rhizoctonia solani partitivirus 2; RsV-717, Rhizoctonia solani virus 717; SLAPV2, Soybean leaf-associated partitivirus 2; SsPV1, Sclerotinia sclerotiorum partitivirus 1; SsPV-S, Sclerotinia sclerotiorum partitivirus S; VCV, Vicia cryptic virus; VdPV1, Verticillium dahliae partitivirus 1; WCCV1, White clover cryptic virus 1; WCCV2, White clover cryptic virus 2. The GenBank accession number of the above-mentioned viruses are listed in Table S2.

The phylogeny of BcPV2 was inferred based on the aa sequences of RdRp and CP of this mycovirus and the known members in *Partitiviridae* (Table S2) using the Maximum-likelihood method. The results showed that BcPV2 belongs to the genus *Alphapartitivirus* (Figure 7A,B). It is closely related to CPV, PmAPV, and SLAPV2, but is distantly related to BcPV1 and BfPV1, which belong to the genera *Betapartitivirus* and *Gammapartitivirus*, respectively.

### 3.6. Horizontal Transmission of BcPV2

**Figure 8:**
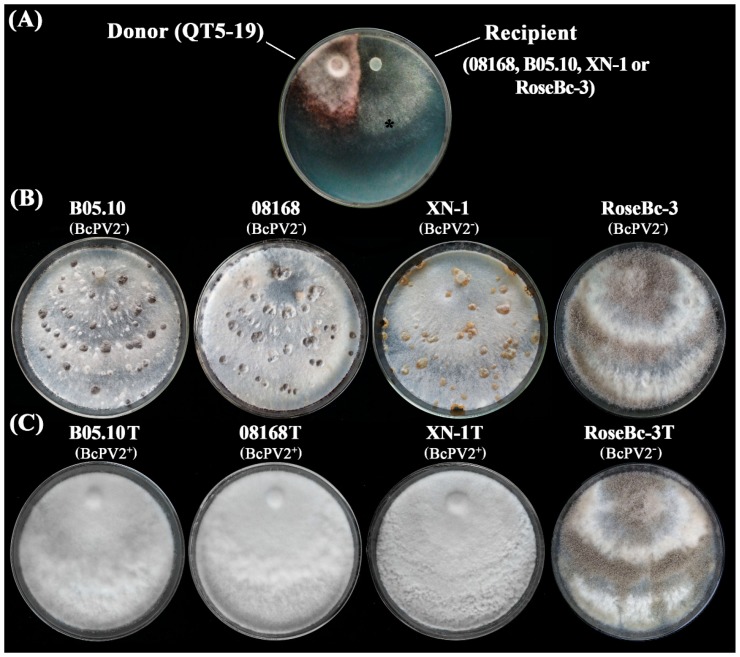
Horizontal transmission of BcPV2 through hyphal contact in pair cultures on PDA. (**A**) A pair culture on PDA with the colonies of isolate QT5-19 (BcPV2 donor, pink color) and a recipient (gray color). The symbol (*) in the colony indicated the area where a mycelial agar plug was removed and transferred to PDA for establishing a BcPV2-transmitted derivative; (**B**) Four PDA cultures (20 °C, 20 day) of the *B. cinerea* isolates free of infection by BcPV2 (BcPV2^−^); (**C**) Four PDA cultures (20 °C, 20 day) of the transfected derivatives of *B. cinerea*. BcPV2^+^, infected by BcPV2, BcPV2^−^, not infected by BcPV2.

**Figure 9:**
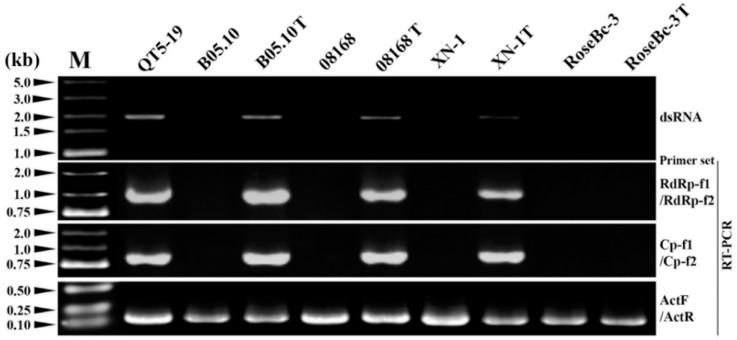
Detection of BcPV2 in different isolates of *Botrytis cinerea* by dsRNA profiling and RT-PCR with specific primers. M = DL5000 DNA marker (TaKaRa). Isolates B05.10T, 08168T, XN-1T, and RoseBc-3T were derived from B05.10, 08168, XN-1 and RoseBc-3 in the pairing cultures of QT5-19/B05.10, QT5-19/08168, QT5-19/XN-1 and QT5-19/RoseBc-3, respectively.

**Figure 10:**
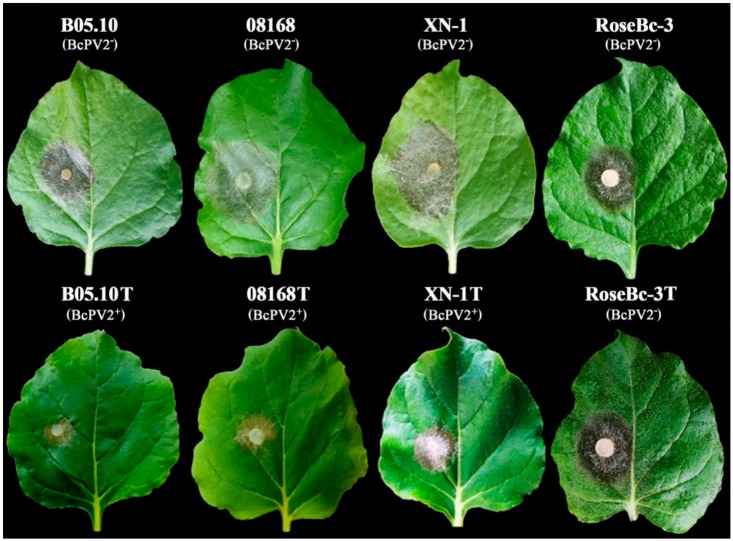
Pathogenicity of different isolates of *Botrytis cinerea* on detached leaves of *Nicotiana benthamiana* (20 °C, 72 h). Note smaller leaf lesion size caused by B05.10T, 08168T, and XN-1T than that caused by B05.10, 08168 and XN-1, whereas similar leaf lesion size caused by RoseBc-3T and RoseBc-3. BcPV^+^ = infected by BcPV2; BcPV2^−^ = not infected by BcPV2.

The pair-culturing technique [23,24,35] was used (Figure 8A) to test horizontal transmission of BcPV2 from the donor isolate QT5-19 to each of the four recipient isolates of *B. cinerea* (08168, B05.10, XN-1, RoseBc-3) on PDA at 20 °C (Figure 8B). Three derivatives (one from each pair culture) were obtained from the colonies of each recipient in the 4-day-old pair cultures of QT5-19 and that recipient. Four representative derivatives (08168T, B05.10T, RoseBc-3T, and XN-1T from 08168, B05.10, RoseBc-3, and XN-1, respectively) were selected (Figure 8C) and compared with their parents for the accumulation of BcPV2 in mycelia, mycelial growth rates and production of conidia/sclerotia on PDA (20 °C), and for pathogenicity on tobacco leaves. Results showed that B05.10T, 08168T, and XN-1T had BcPV2 accumulation, whereas RoseBc-3T did not have any detectable BcPV2 accumulation (Figure 9; Table 2). Therefore, horizontal transmission of BcPV2 was successful from QT5-19 to three of the four investigated isolates, but was unsuccessful from QT5-19 to RoseBc-3. Results also showed that B05.10T, 08168T, and XN-1T grew at the average rate of 1.5 cm/d, which did not significantly differ (*p* > 0.05) from those of their parents. However, they failed to produce conidia and sclerotia after incubation at 20 °C for 20 days, and these features differed from their parents (Table 2). As expected, RoseBc-3T showed the same phenotype as its parental isolate RoseBc-3 both in growth rate and in the production of conidia and sclerotia (Table 2). On tobacco leaves, B05.10T, 08168T and XN-1T caused average leaf lesion diameters of 2.2, 2.3, and 2.9 cm (20 °C, 3 dpi), respectively, and the values were significantly (*p* < 0.05) smaller than those of B05.10, 08168 and XN-1, which caused lesion diameters of 3.1, 3.8, and 4.0 cm, respectively (Table 2, Figure 10). However, RoseBc-3T caused an average lesion diameter of 2.2 cm, not significantly different (*p* > 0.05) from the average leaf lesion caused by RoseBc-3 (2.3 cm in diameter) (Table 2, Figure 10). Therefore, the introduction of BcPV2 from QT5-19 to B05.10, 08168, and XN-1 caused debilitation of pathogenicity and deficiency in formation of conidia and sclerotia.

## 4. Discussion

This study demonstrated that the pink isolate QT5-19 of *B. cinerea* is a hypovirulent isolate on apple, cucumber, oilseed rape, strawberry, table grapes, tobacco, and tomato, compared to the gray isolates 08168, B05.10, and XN-1of *B. cinerea*, which aggressively infected tobacco. Botrytis cinerea partitivirus 2 (BcPV2) was identified in QT5-19, whereas BcPV2 and other RNA mycoviruses were not detected in 08168, B05.10 and XN-1. Moreover, isolates 08168T, B05.10T and XN-1T infected by BcPV2 in the horizontal transmission experiment showed a significant (*p* < 0.05) decrease in pathogenicity on tobacco, compared to their parents (08168, B05.10, XN-1). This result suggests that BcPV2 is closely associated with hypovirulence of *B. cinerea*. Previous studies have reported several mycoviruses-infected hypovirulent isolates in B. cinerea, including CanBc-1 (infested with BcMV1), CCg378 (infested with BfPV1), BerBc-1 (infested with BcRV1), Ecan17-2 (infested with BcMyV1), and HBtom-372 (infested with BcFV1 and BcHV1) [9,23,27,28,31,32]. QT5-19 grew almost as rapidly as 08168, B05.10 and XN-1 on PDA at 20 °C, but failed to produce conidia and sclerotia. These cultural features differ from those in CanBc-1, Ecan17-2, and HBtom-372, which grew slowly on PDA [23], and from those of BerBc-1, which grew rapidly and formed sclerotia on PDA [32]. Moreover, the BcPV2-transfected isolates 08168T, B05.10T, and XN-1T also showed vigorous growth on PDA and the defect in production of conidia and sclerotia. These results suggest that BcPV2 in QT5-19 as well as in 08168T, B05.10T, and XN-1T may have no negative effect on vegetative growth of these isolates of *B. cinerea*; however, it has attenuation effects on pathogenicity, as well as on production of conidia and sclerotia. Further transcriptomic analysis of the isogenic BcPV2-free and BcPV2-infected isolates is necessary to determine the BcPV2-responsive genes associated with (or responsible for) pathogenicity, and production of conidia and sclerotia in *B. cinerea*.

The viruses in the family *Partitiviridae* have been reported in fungi as well as in plants and protozoa [13]. There are at least five genera in this family, namely *Alphapartitivirus*, *Betapartitivirus*, *Cryspovirus*, *Gammapartitivirus* and *Deltapartitivirus* according to the recent report in ICTV (https://talk.ictvonline.org). Previous studies showed that most mycoviruses in *Partitiviridae* have no deleterious effects on their fungal hosts [4] and only a few partitiviruses were found to be associated with abnormal phenotypes in fungi. For example, Rhizoctonia solani partitivirus 2 (RsPV2) is closely associated with hypovirulence in *Rhizoctonia solani*, the causal agent of rice sheath blight [47]. Sclerotinia sclerotiorum partitivirus 1 (SsPV1) can confer hypovirulence in *S. sclerotiorum* and related species [48]. Two partitiviruses, namely Botrytis cinerea partitivirus 1 (BcPV1) and Botryotinia fuckeliana partitivirus 1 (BfPV1), have been reported in *B. cinerea* [31,34]. BcPV1 was found to be associated with hypovirulence of *B. cinerea* [31], whereas the effect of BfPV1 on pathogenicity of *B. cinerea* remains unknown. Moreover, Xiao and colleagues (2014) reported that introduction of the virus particles of SsPV1 to *B. cinerea* caused decrease in mycelial growth and pathogenicity of *B. cinerea*, but caused increase in conidial production by this fungus [48]. Phylogenetic analysis in this study showed that BcPV2 is a unique member in *Alphapartitivirus* and it is phylogenetically distant from BcPV1 in *Betapartitivirus*, SsPV1 in *Betapartitivirus,* and BfPV1 in *Gammapartitivirus*. The close association between BcPV2 infection and hypovirulence observed in this study provides an example of *Alphapartitivirus*-mediated hypovirulence in *B. cinerea*.

As mentioned above, QT5-19 infected with BcPV2 hardly infected tobacco and six other crops. BcPV2 in QT5-19 was successfully transmitted to three other isolates of *B. cinerea* (08168, B05.10, XN-1) through hyphal contact (possibly through anastomosis). The resulting derivatives B05.10T, XN-1T, and 08168T infested with BcPV2 grew rapidly on PDA at 20 °C, but failed to produce conidia and sclerotia. These results suggest that BcPV2 in B05.10T, XN-1T and 08168T has a significant attenuation effect on the formation of conidia and sclerotia. However, the pathogenicity test showed that B05.10T, XN-1T, and 08168T could infect tobacco and caused formation of necrotic leaf lesions (Figure 10), although the average leaf lesion diameters caused by these BcPV2-infected derivatives were significantly (*p* < 0.05) smaller than those caused by their parents (reduced by 27% to 39%). This result suggests that BcPV2 in QT5-19 may have a larger pathogenicity-attenuation effect than it does in B05.10T, XN-1T, and 08168T. Different genetic backgrounds in isolates QT5-19, B05.10T, XN-1T and 08168T might be responsible for this phenomenon.

Previous studies showed that it is difficult to use the RNA mycoviruses such as BcMV1, BcFV1, BcHV1, BcRV1, and BcMyV1 in the corresponding hypovirulent isolates to control virulent isolates of *B. cinerea* [9,23,28,32]. There are two possible reasons: (*i*) limited horizontal transmissibility of the mycoviruses possibly due to hyphal incompatibility between the mycoviruses-infected hypovirulent isolates and virulent isolates; and (*ii*) low competitiveness of the mycoviruses-infected hypovirulent isolates due to poor mycelial growth. The hypovirulent isolate QT5-19 grew almost as rapidly as the virulent isolates 08168, B05.10, and XN-1 on PDA, suggesting that it may have a strong competitive ability. Meanwhile, BcPV2 in QT5-19 (donor) was successfully transmitted to four of the five investigated recipient isolates, suggesting that BcPV2 in QT5-19 may have a wider horizontal transmission spectrum than the mycoviruses reported in previous studies [9,23,28,32]. Therefore, QT5-19 with BcPV2 may have potential to be exploited as a biocontrol agent against virulent individuals of *B. cinerea*. Additional studies on evaluation of the biocontrol efficacy of QT5-19 are warranted.

In conclusion, this study found that the hypovirulent isolate QT5-10 of *B. cinerea* has distinct cultural characteristics (rapid mycelial growth, but with defects in formation of conidia and sclerotia). A novel partitivirus in *Alphapartitivirus*, Botrytis cinerea partitivirus 2 (BcPV2), was identified in QT5-19. BcPV2 was successfully transmitted from QT5-19 to virulent isolates 08168, B05.10, and XN-1, and the resulting BcPV2-transfected derivatives were attenuated both in pathogenicity and in production of conidia and sclerotia.

## Figures and Tables

**Table 1:** Summary of the results in the BLASTp search for RNA-dependent RNA polymerase (RdRp) and coat protein (CP) of *Botrytis cinerea* partitivirus 2 (BcPV2).

Viruses	GenBank Acc. No.	Size (aa)	Identity (%)	Overlap (Positions)	Bit Score	E-Value
RdRp						
Ceratobasidium partitivirus (CPV)	AOX47571	579	76	440/578	917	0.0
Soybean leaf-associated partitivirus 2 (SLAPV2)	ALM62247	602	58	341/583	681	0.0
Grapevine partitivirus (GPV)	AFX73022	584	53	311/587	631	0.0
Powdery mildew-associated partitivirus (PmAPV)	YP_009272944	584	51	301/587	605	0.0
Sclerotinia sclerotiorum partitivirus S (SsPV-S)	YP_003082248	580	46	263/566	472	1 × 10^−157^
Rhizoctonia solani partitivirus 1 (RsPV1)	AND83003	603	43	234/549	431	4 × 10^−141^
Flammulina velutipes isometric virus (FvIV)	BAH08700	587	42	252/594	436	2 × 10^−143^
Fusarium solani partitivirus 2 (FsPV2)	BAQ36631	608	41	242/587	402	5 × 10^−130^
Rosellinia necatrix partitivirus 5 (RnPV5)	BAM36403	647	40	230/568	414	6 × 10^−134^
Botryotinia fuckeliana partitivirus 1 (BfPV1)	YP_001686789	540	13 *	40/293	- **	-
CP						
Ceratobasidium partitivirus (CPV)	AOX47604	370	60	221/370	473	1 × 10^−161^
Soybean leaf-associated partitivirus 2 (SLAPV2)	ALM62248	496	34	169/498	272	8 × 10^−82^
Powdery mildew-associated partitivirus (PmAPV)	YP_009272945	527	31	164/528	238	2 × 10^−68^
Diuris pendunculata cryptic virus (DpCV)	AFY23215	496	31	36/116	58	2 × 10^−05^
Carrot cryptic virus (CCV)	ACL93279	490	26	38/147	45.1	0.33
Spinach cryptic virus 1 (SCV)	APX42422	488	24	44/181	48.5	0.023
Rosellinia necatrix partitivirus 2 (RnPV2)	YP_007419078	483	23	77/333	47.4	0.057
Arabidopsis halleri partitivirus 1 (AhPV1)	YP_009273019	487	22	72/330	57	6 × 10^−05^
Botrytis cinerea partitivirus 1 (BcPV1) *	AGQ21570	634	14 *	56/389	-	-
Botryotinia fuckeliana partitivirus 1 (BfPV1) *	YP_001686790	436	10 *	41/408	-	-

* The values were calculated using the DNAMAN software (version 7.0), rather than provided in BLASTp search in NCBI, as the identity level between BcPV2 and BcPV1/BfPV1 is too low. ** The values cannot be calculated in the analysis with DNAMAN.

**Table 2:** Horizontal transmission of BcPV2 from QT5-19 to isolates of *B. cinerea* (B05.10, 08168, RoseBc-3, XN-1) and effect of BcPV2 infection on mycelial growth, conidial production, sclerotial production, and pathogenicity of the recipients.

Isolate	BcPV2 ^x^	Mycelial Growth Rate (cm/day) ^y^	Conidial Production ^y^	No. Sclerotia per Dish ^y^	Lesion Diameter (cm) ^z^
QT5-19	+	1.2	-	0	0.2
B05.10	-	1.5	+	36	3.1
B05.10T	+	1.5	-	0 **	2.2 *
08168	-	1.5	+	38	3.8
08168T	+	1.5	-	0 **	2.3 *
XN-1	-	1.5	+	37	4.0
XN-1T	+	1.5	-	0 **	2.9 *
RoseBc-3	-	1.4	+	25	2.3
RoseBc-3T	-	1.4	+	22	2.2

^x^ BcPV2 was detected by dsRNA profiling and RT-PCR using the specific primer pairs RdRp-f1/RdRp-r1 (dsRNA 1) and CP-f1/CP-r1 (dsRNA-2). “+”, the presence of BcPV2; “-”, the absence of BcPV2. ^y^ Mycelial growth rate, conidial production, and sclerotial production were determined on PDA at 20 °C. Each value is an average of 5 replicates. “+” with conidia, “-” without conidia. “**” indicates significant difference at *p* < 0.01 between each recipient and its transfected derivative according to Student’s *t* test. ^z^ Pathogenicity was determined on detached tobacco leaves (20 °C, 72 h). Each value of lesion diameter is an average of seven replicates. “*” indicates significant difference at *p* < 0.05 between each recipient and its transfected derivative isolate according to Student’s *t* test.

## Supplementary Materials

The following are available online at http://www.mdpi.com/1999-4915/11/1/24/s1, Figure S1: Phenotypic and molecular characterization of bikaverin biosynthesis in the pink-colored isolate QT5-19 of *Botrytis cinerea* (A) Difference between QT5-19 and B05.10 in accumulation of the pink pigment on potato dextrose agar (20 °C) (B) RT-PCR detection of expression of the genes (*bcbik1* to *bcbik6*) responsible for biosynthesis of the pink pigment bikaverin. The primers for related genes used in RT-PCR are listed in Table S1. (C) A schematic diagram showing the bikaverin-biosynthesis gene cluster in isolates QT5-19 and B05.10. The gray-colored region indicates the absence of *bcbik1* in B05.10. Arrows indicate the direction of gene transcription. Figure S2: Alignment of the partial nucleotide sequences of *bcbik1* coding for polyketide synthase (PKS) in isolates QT5-19 and 1750 of *Botrytis cinerea*, and the homologs of *bcbik1* in *Fusarium oxysporum* and *Fusarium anthophilum*. Figure S3: Pathogenicity of isolates QT5-19 and B05.10 of *B. cinerea* on fruits of tomato, table grape and apple, and on leaves of cucumber, rapeseed and strawberry. Figure S4: A schematic diagram showing the strategy for cDNA cloning of the full-length sequences of the two dsRNAs in Botrytis cinerea partitivirus 2 (BcPV2). Figure S5: Northern-blotting detection of the dsRNAs extracted from the mycelia of *Botrytis cinerea* QT5-19. Figure S6: Detection of BcPV2 in different isolates of *Botrytis cinerea* by dsRNA profiling and RT-PCR. Table S1: List of the oligonucleotide primers or adaptor used in this study. Table S2: List of partitiviruses for multiple sequence alignment and phylogenetic analysis.
