# Supplementary material for: A Novel Partitivirus in the Hypovirulent Isolate QT5-19 of the Plant Pathogenic Fungus Botrytis cinerea
Source: Viruses. 2019 Jan 3;11(1):24. doi: 10.3390/v11010024 (PMC6356794; doi:10.3390/v11010024)
Supplement: Supplementary file 1 [file viruses-11-00024-s001.pdf]

viruses

Supporting Information

A Novel Partitivirus in the Hypovirulent Isolate QT5-19 of the Plant Pathogenic Fungus *Botrytis cinerea*

Kamaruzzaman[1], Guoyuan He[1], Mingde Wu[1], Jing Zhang[1], Long Yang[1], Weidong Chen[2] and Guoqing Li[1*]

[1] The Key Laboratory of Plant Pathology of Hubei Province and The State Key Laboratory of Agricultural Microbiology, Huazhong Agricultural University, Wuhan 430070, China.
[2] U. S. Department of Agriculture, Agricultural Research Service, Washington State University, Pullman, WA, 99164, USA.
* Correspondence: guoqingli@mail.hzau.edu.cn

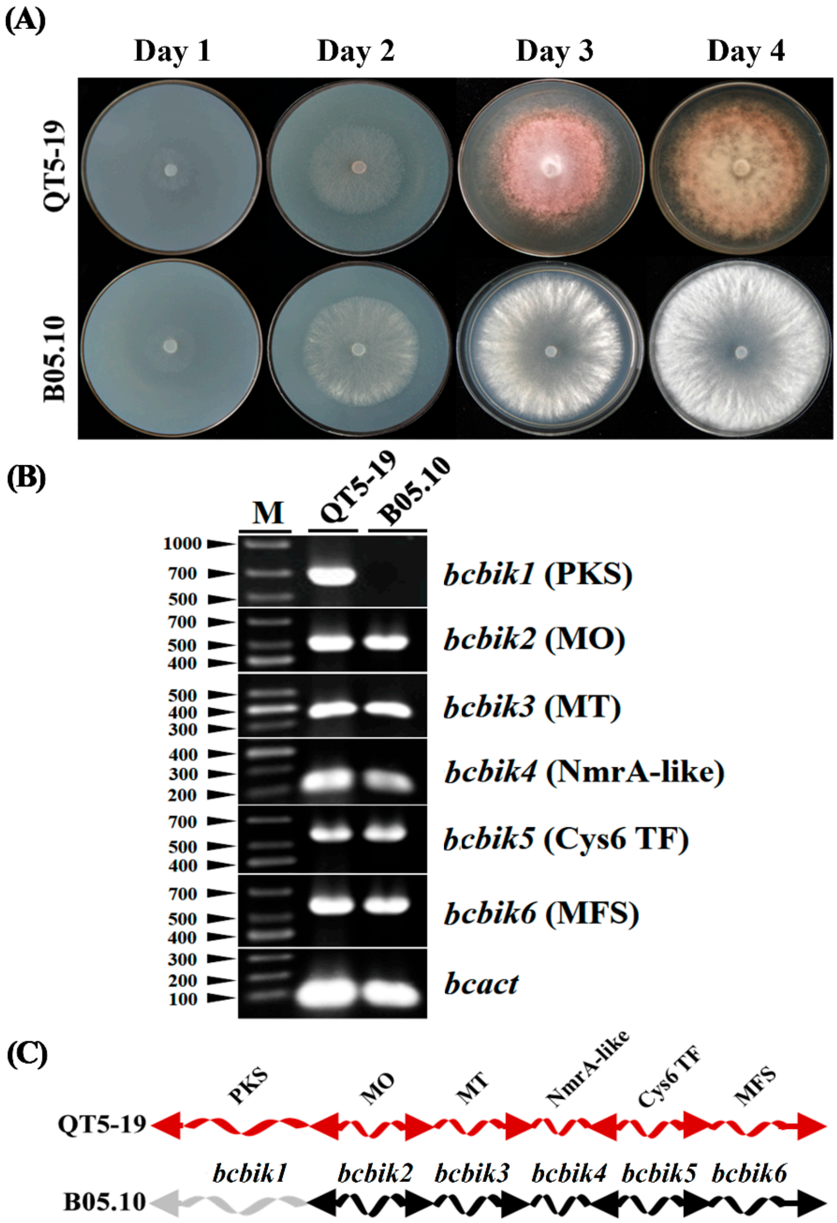

Figure S1. Phenotypic and molecular characterization of bikaverin biosynthesis in the pink-colored isolate QT5-19 of *Botrytis cinerea*. (A) Difference between QT5-19 and B05.10 in accumulation of the pink pigment on potato dextrose agar (20ºC). (B) RT-PCR detection of expression of the genes (*bcbik1* to *bcbik6*) responsible for biosynthesis of the pink pigment bikaverin. The primers for related genes used in RT-PCR are listed in Table S1. (C) A schematic diagram showing the bikaverin-biosynthesis gene cluster in isolates QT5-19 and B05.10. The gray-colored region indicates the absence of *bcbik1* in B05.10. Arrows indicate the direction of gene transcription.

Alignment of the nucleotide sequences of *Bcbik1* and its homologs

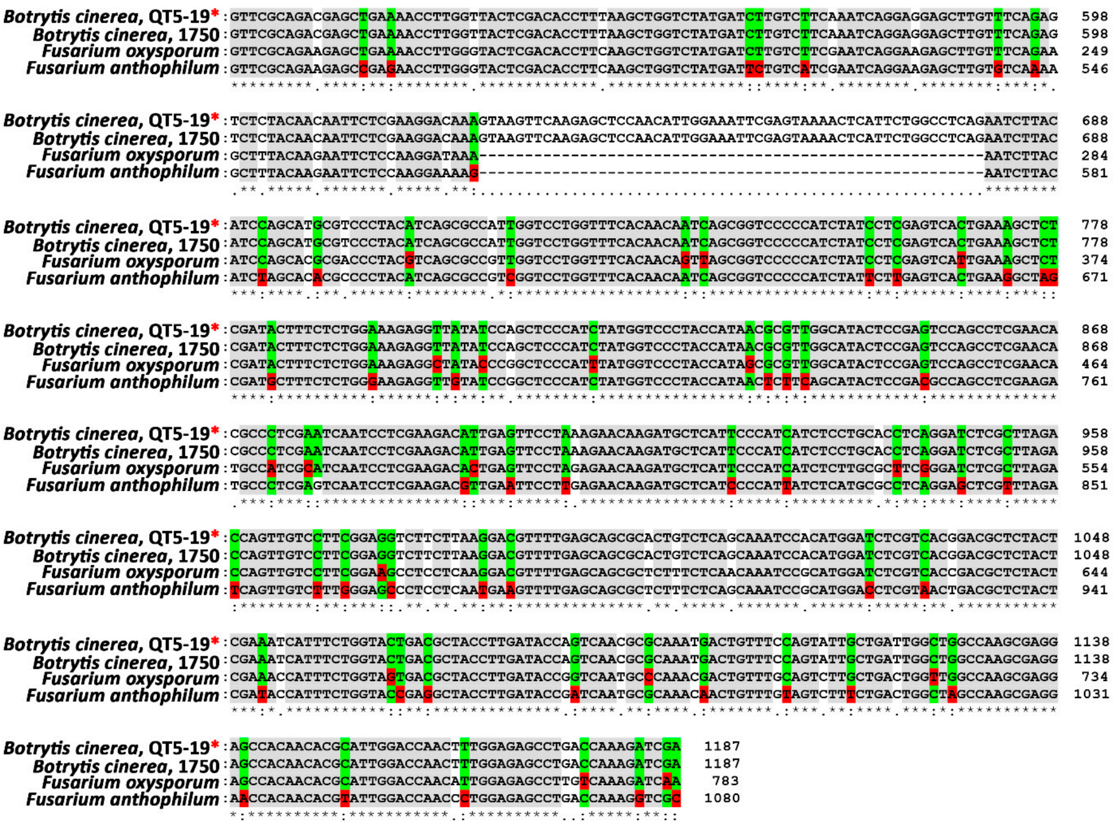

Figure S2. Alignment of the partial nucleotide sequences of *bcbik1* coding for polyketide synthase (PKS) in isolates QT5-19 and 1750 of *Botrytis cinerea*, and the homologs of *bcbik1* in *Fusarium oxysporum* and *Fusarium anthophilum*. The nucleotide sequences were aligned using CLUSTAL_W. "*", identical nucleotides; "-", deleted nucleotides. GenBank Acc. Nos. for *bcbik1* and its homologs: MH747471 for *B. cinerea* isolate QT5-19, HE802550 for *B. cinerea* of 1750, XM_018382794 for *F. oxysporum*, and KU179878 for *F. anthophilum*.

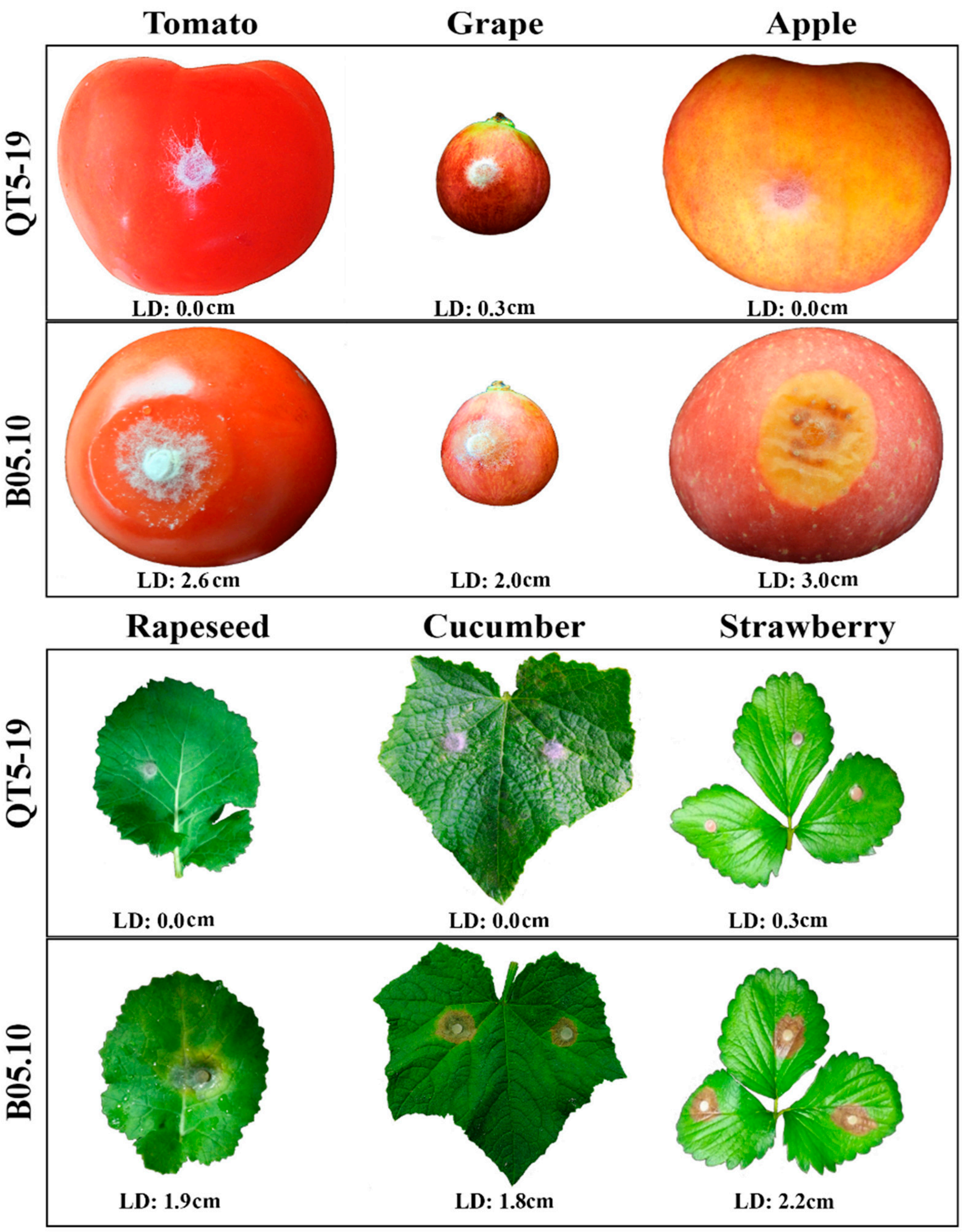

Figure S3. Pathogenicity of isolates QT5-19 and B05.10 of *B. cinerea* on fruits of tomato, table grapes and apple, and on leaves of cucumber, rapeseed and strawberry. Mycelial agar plugs (5 mm) of QT5-19 and B05-10 were placed on the surface of the fruits and leaves, which were kept at 20ºC under humid conditions for 3 days. LD = lesion diameter (on average).

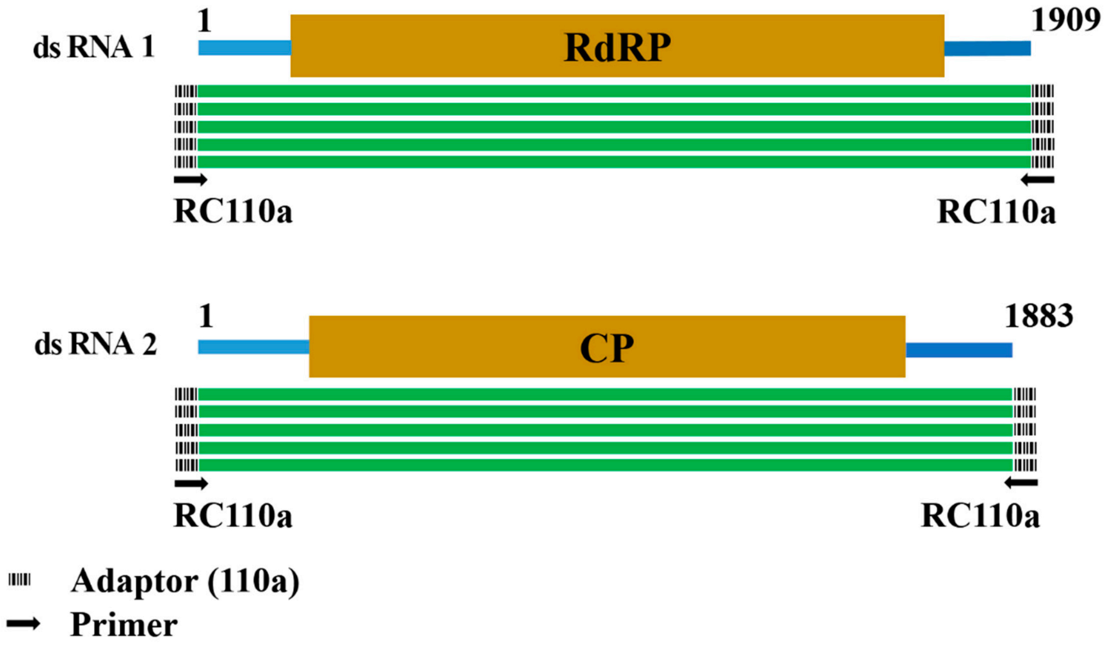

Figure S4. A schematic diagram showing the strategy for cDNA cloning of the full-length sequences of the two dsRNAs in Botrytis cinerea partitivirus 2 (BcPV2). The adaptor/primers and their nucleotide sequences were listed in Table S1.

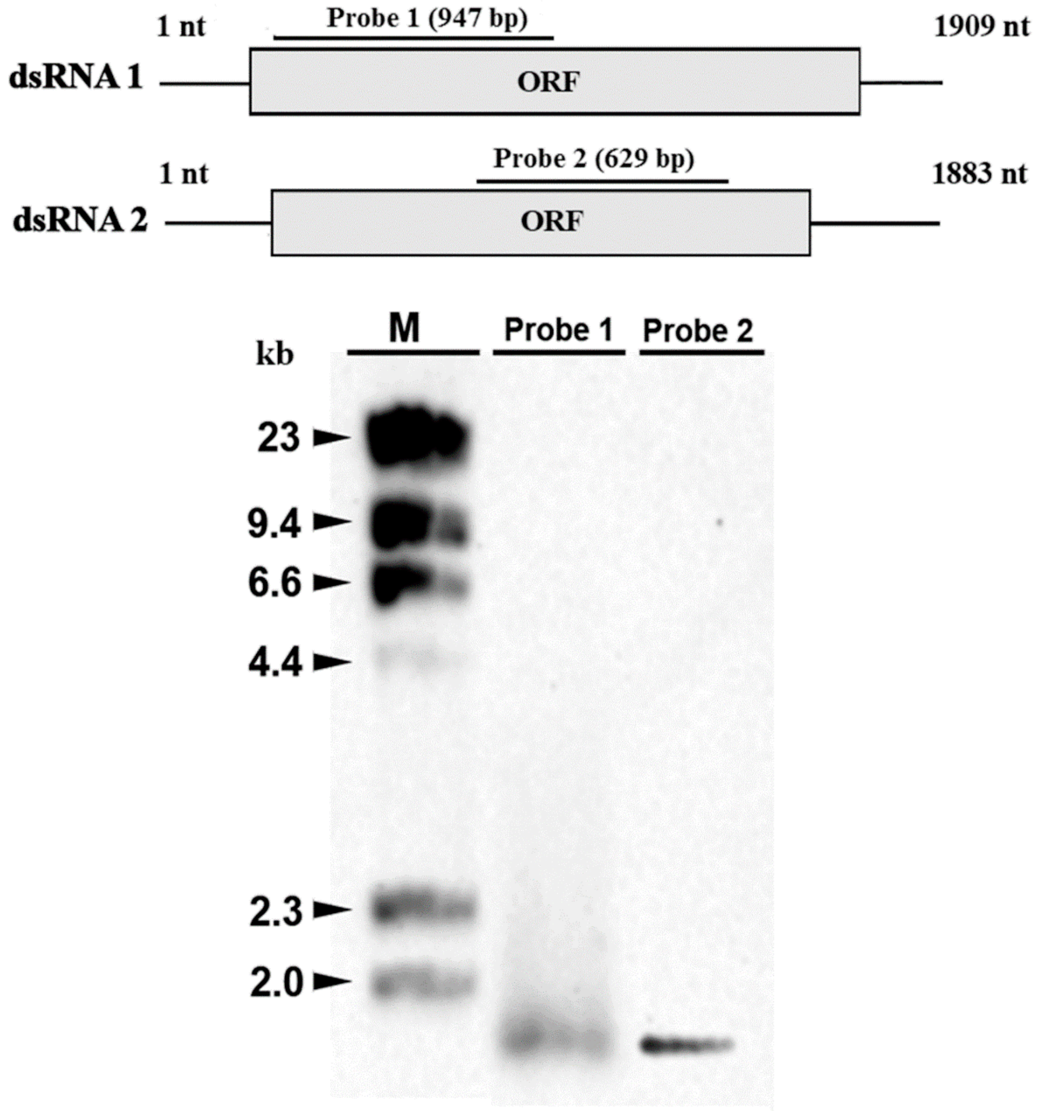

Figure S5. Northern-blotting detection of the dsRNAs extracted from the mycelia of *Botrytis cinerea* QT5-19. The position of the two DNA probes in the genome of BcPV2 was shown on the top.

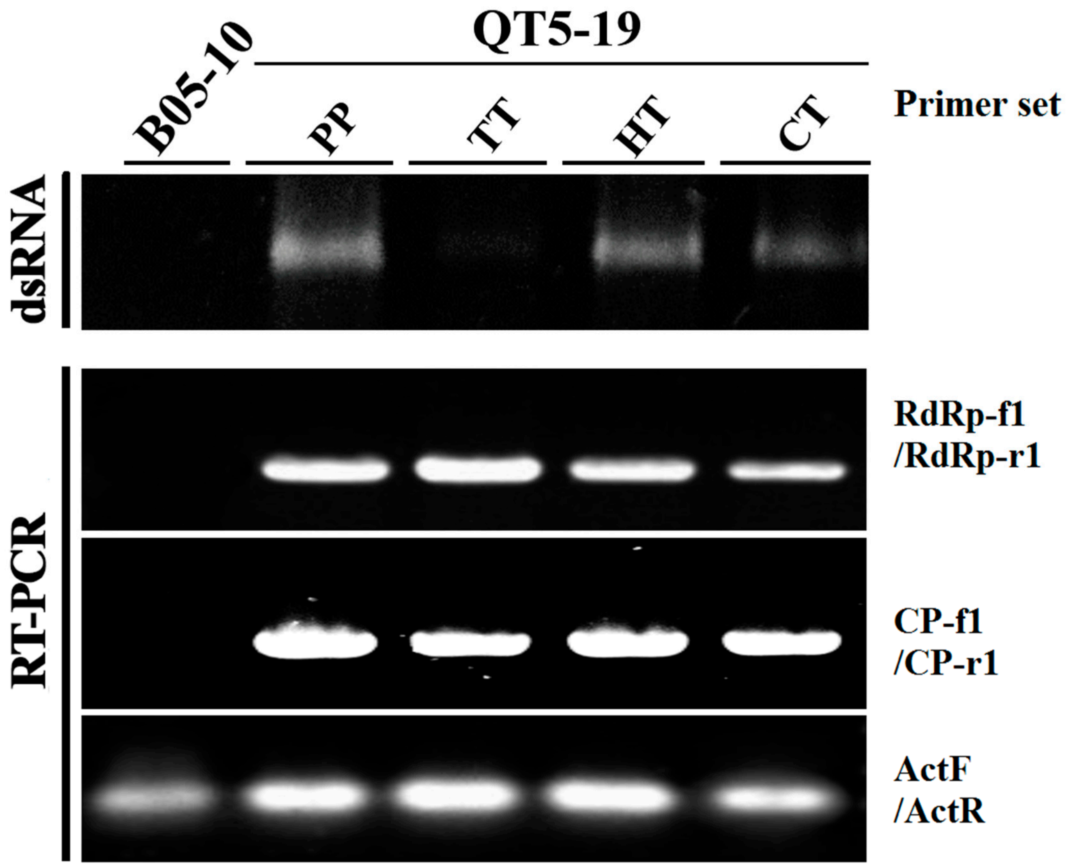

Figure S6. Detection of BcPV2 in different isolates of *Botrytis cinerea* by dsRNA profiling and RT-PCR. Four derivatives of QT5-19 from protoplast regeneration (PP), thermal therapy (TH), hyphal tipping (HT) and chemical therapy (CT). In dsRNA profiling, the dsRNA extracts from different isolates were treated with DNase I and S1 nuclease before loading for agarose gel electrophoresis. In RT-PCR, the actin gene *bcact* amplified by the primers ActF/ActR was used as control. The oligonucleotide sequences of the primers were shown in Supplementary Table S1.

Table S1. List of the oligonucleotide primers or adaptor used in this study.

Primer name	Sequence (5´to 3´)	Size of the product
To confirm the taxonomic status of isolates 08168, B05.10, QT5-19, RoseBc-3 and XN-1 as *B. cinerea*		
Bc-f	CAGGAAACACTTTTGGGGATA	327 bp
Bc-r	GAGGGACAAGAAAATCGACTAA	
To detect and clone the ITS region		
ITS1	TCCGTAGGTGAACCTGCGG	539 bp
ITS4	TCCTCCGCTTATTGATATGC	
To synthesize cDNAs of BcPV2		
110a	(PO4)-TATCTTATCGGCGTGTCCCCC-(NH2)	1930 bp for dsRNA1
RC110a	GGGGGACACGCCGATAAGATA	1904 bp for dsRNA2
To detect the cDNA insert in the plasmid pMD18-T		
M13F-47	CGCCAGGGTTTTCCCAGTCACGAC	1930 bp for dsRNA1
M13R-48	AGCGGATAACAATTTCACACAGGA	1904 bp for dsRNA2
To detect BcPV2/QT5-19 in RT-PCR		
RdRp-f1	GTTCCGACCTCATCGTCGTT	970 bp
RdRp-r1	TGCTTCGGAGGTAAGGGTTG	
CP-f1	CCGTTCCTTTTACACGCCAG	796 bp
CP-r1	ACGTGTGACAGTAGACGCTG	
To prepare the DNA probes used in Northern blotting for detection of the BcPV2 dsRNAs		
RdRp-f2	GATCAATCGACGTACCAAA	946 bp

RdRp-r2	AAGAGCAGATTGACCGAC	
CP-f2<br>CP-r2	ACGACTCCCGTTCCTTT<br>TTCTTCAGTTTCGGTTGG	629 bp
To detect expression of *bcbik1* in QT5-19 and B05.10		
Bcbik1-f<br>Bcbik1-r	GTTCGCAGACGAGCTGAAA<br>TCGATCTTTGGTCAGGCTCT	679 bp
To detect expression of *bcbik2* in QT5-19 and B05.10		
Bcbik2-f<br>Bcbik2-r	TGGCGATGCAGCTTGTAAGA<br>GACTCGCAAACTCATTCCGC	503 bp
To detect expression of *bcbik3* in QT5-19 and B05.10		
Bcbik3-f<br>Bcbik3-r	TTGCGTCTATGGTTACCGCT<br>AAGGCTTCGGCTAGCTTCAC	348 bp
To detect expression of *bcbik4* in QT5-19 and INRA 1750		
Bcbik4-f<br>Bcbik4-r	TTCATCTACGCCGTACGCTC<br>TGTGGCACCTCGAGTTCTTC	212 bp
To detect expression of *bcbik5* in QT5-19 and B05.10		
Bcbik5-f<br>Bcbik5-r	GGATGCCATCGCTTCTCTGA<br>TCACACCTGACCAACGTGAG	570 bp
To detect expression of *bcbik6* in QT5-19 and B05.10		
Bcbik6-f<br>Bcbik6-r	ATCTGCCTGCTGCTTACGAT<br>AAGATGGTGGTGAAGCCAAC	572 bp

Note: The primer pair ActF/ActR was designed based on the actA gene in *Botryotinia fuckeliana* (GenBank Acc. No. AJ000335.1). The primer set for RT-PCR detection of the expression of *bcbik1*was adopted from Schumacher and colleagues (2013) (Schumacher *et al*., 2013. *PLoS ONE* 8, e53729). The primer sets for RT-PCR detection of *bcbik2*, *bcbik3*, *bcbik4*, *bcbik5, bcbik6* were designed based on the DNA sequences of these genes under the GenBank accession numbers HE802545, HE802546, HE802547, HE802548, HE8025449, respectively.

Table S2. List of partitiviruses for multiple sequence alignment and phylogenetic analysis.

Name	GenBank Acc. No.		Genus
	dsRNA 1	dsRNA 2	
Botrytis cinerea Partitivirus 2 (BcPV2)★	MG011707	MG011708	*Alphhapartitivirus*
Beet cryptic virus 1(BCV1)	ACA81389	ACA81390	
Carrot cryptic virus (CCV)	ACL93278	ACL93279	
Ceratobasidium partitivirus (CPV)	AOX47571	AOX47604	
Cherry chlorotic rusty spot associated partitivirus (CCRSaPV)	CAH03668	CAH03669	
Chondrostereum purpureum cryptic virus 1 (CpCV1)	CAQ53729	CAQ53730	
Dill clover cryptic virus 1 (DCV1)	KF484726	AGY36137	
Diuris pendunculata cryptic virus (DpCV)	AFQ95555	AFY23215	
Flammulina velutipes browning virus (FvBV)	BAH56481	BAH56482	
Helicobasidium mompa partitivirus V70 (HmPVV70)	BAC23065	-	
Heterobasidion partitivirus 1 (HetPV1)	ADV15441	ADV15442	
Heterobasidion partitivirus 12 (HetPV12)	AHL25151	AHL25152	
Heterobasidion partitivirus 13 (HetPV13)	AHL25153	AHL25154	
Heterobasidion partitivirus 15 (HetPV15)	AHL25162	AHL25163	
Heterobasidion partitivirus 3 (HetPV3)	ACO37245	ACO37246	
Powdery mildew-associated partitivirus (PmAPV)	AOF47283	AOF47284	
Rhizoctonia solani partitivirus 2 (RsPV2)	AGY54938	AGY54939	
Rosellinia necatrix partitivirus 2 (RnPV2)	BAM78602	BAK53192	
Sclerotinia sclerotiorum partitivirus S (SsPV-S)	ACT55329	ACT55330	
Soybean leaf-associated partitivirus 2 (SLAPV2)	ALM62247	ALM62248	
Vicia cryptic virus (VCV)	EF173389	EF173390	
White clover cryptic virus 1 (WCCV1)	AAU14888	AAU14889	
Atkinsonella hypoxylon partitivirus (AhV)	AAA61829	AAA61830	*Betapartitivirus*
Botrytis cinerea Partitivirus 1 (BcPV1)	-	AGQ21570	
Cannabis cryptic virus (CaCV)	AET80948	AET80949	
Dill cryptic virus 2 (DCV2)	AGJ83771	AGJ83772	
Fusarium poae virus 1 (FpV1)	NP_624349	NP_624348	
Grapevine partitivirus (GPV)	AFX73019	-	
Heterobasidion partitivirus 2 (HetRV2)	ADL66905	ADL66906	
Heterobasidion partitivirus 8 (HetRV8)	AFW17810	AFW17811	
Pleurotus ostreatus virus 1 (PoV1)	AAT07072	AAT06080	
Rhizoctonia solani virus 717 (RsV-717)	AAF22160	AAF40300	
Rosellinia necatrix partitivirus 1 (RnV1)	BAD98237	BAD98238	
Sclerotinia sclerotiorum partitivirus 1 (SsPV1)	AFR78160	AFR78159	
White clover cryptic virus 2 (WCCV2)	AGJ83763	AGJ83764	
Botryotinia fuckeliana partitivirus 1 (BfPV1)	YP_001686789	YP_001686790	*Gammapartitivirus*
Discula destructiva virus 1 (DdV1)	AAG59816	AAK13165	
Discula destructiva virus 2 (DdV2)	AAK59379	AAK59380	
Fusarium solani virus 1 (FsV1)	BAA09520	BAA09521	
Ophiostoma partitivirus 1 (OPV1)	CAJ31886	CAJ31887	
Penicillium stoloniferum virus F (PsV-F)	AAU95758	AAU95759	
Penicillium stoloniferum virus S (PsV-S)	AAN86834	AAN86835	
Verticillium dahliae partitivirus 1 (VdPV1)	AGI52210	AGI52209	
Beet cryptic virus 2 (BCV2)	ADP24757	AGQ49466	*Deltapartitivirus*
Fig cryptic virus (FCV)	CBW77436	CBW77437	
Fragaria chiloensis cryptic virus (FcCV)	AAZ06131	ABC73696	
Pepper cryptic virus 1 (PCV1)	AEJ07890	AEJ07891	

Pepper cryptic virus 2 (PCV2)	AEJ07892	AEJ07893
Persimmon cryptic virus (PeCV)	CCH50609	CCH50610
Raphanus sativus cryptic virus 2 (RsCV2)	YP_001686783	YP_001686784
Raphanus sativus cryptic virus 3 (RsCV3)	YP_002364401	YP_002364402
Rose cryptic virus 1 (RoCV1)	YP_001686786	YP_001686787

Note: The symbol "-" indicates that accession number is not available in GenBank.
